# Smoking mediates the relationship between SES and brain volume: The CARDIA study

**DOI:** 10.1371/journal.pone.0239548

**Published:** 2020-09-21

**Authors:** Ryan J. Dougherty, Justine Moonen, Kristine Yaffe, Stephen Sidney, Christos Davatzikos, Mohamad Habes, Lenore J. Launer

**Affiliations:** 1 Johns Hopkins University, Baltimore, Maryland, United States of America; 2 National Institute on Aging, Bethesda, Maryland, United States of America; 3 University of California, San Francisco, San Francisco, California, United States of America; 4 San Francisco VA Health Care System, San Francisco, California, United States of America; 5 Kaiser Permanente Division of Research, Oakland, California, United States of America; 6 Kaiser Permanente of Northern California, Oakland, California, United States of America; 7 University of Pennsylvania, Philadelphia, Pennsylvania, United States of America; Nathan S Kline Institute, UNITED STATES

## Abstract

**Objective:**

Investigate whether socioeconomic status (SES) was related to brain volume in aging related regions, and if so, determine whether this relationship was mediated by lifestyle factors that are known to associate with risk of dementia in a population-based sample of community dwelling middle-aged adults.

**Methods:**

We studied 645 (41% black) participants (mean age 55.3±3.5) from the Coronary Artery Risk Development in Young Adults (CARDIA) study who underwent brain magnetic resonance imaging. SES was operationalized as a composite measure of annual income and years of education. Gray matter volume was estimated within the insular cortex, thalamus, cingulate, frontal, inferior parietal, and lateral temporal cortex. These regions are vulnerable to age-related atrophy captured by the Spatial Pattern of Atrophy for Recognition of Brain Aging (SPARE-BA) index. Lifestyle factors of interest included physical activity, cognitive activity (e.g. book/newspaper reading), smoking status, alcohol consumption, and diet. Multivariable linear regressions tested the association between SES and brain volume. Sobel mediation analyses determined if this association was mediated by lifestyle factors. All models were age, sex, and race adjusted.

**Results:**

Higher SES was positively associated with brain volume (β = .109 SE = .039; *p* < .01) and smoking status significantly mediated this relationship (z = 2.57). With respect to brain volume, smoking accounted for 27% of the variance (β = -.179 SE = .065; *p* < .01) that was previously attributed to SES.

**Conclusion:**

Targeting smoking cessation could be an efficacious means to reduce the health disparity of low SES on brain volume and may decrease vulnerability for dementia.

## Introduction

Low socioeconomic status (SES) has been linked to a variety of negative lifestyle factors [1–5] and greater risk of developing dementia [6–10]. It has been well documented that pathophysiological processes of dementia, including brain atrophy, begin to develop during midlife, decades prior to cognitive impairment [11–13]. Importantly, lifestyle factors associated with SES have shown to influence brain volume [14–16] and dementia risk [17–19]. Recent research suggests SES may be related to alterations in brain health [20–22], however, prior studies have not investigated modifiable lifestyle factors that may contribute to the observed associations.

Epidemiological cohort studies have identified specific brain regions vulnerable to early age-related atrophy including the insular cortex, thalamus, cingulate cortex, frontal inferior parietal, and lateral temporal cortex [14, 15]. Investigating whether SES is associated with brain volume in a community-based middle age population will provide novel epidemiological health information. Further, examining lifestyle factors which may contribute to this relationship is particularly relevant as midlife is a period of aging when behaviors may be modified to decrease one’s risk of dementia [17–19]. There is a need to identify modifiable lifestyle factors that may be highest yield when targeted early to mitigate the deleterious health effects of SES disparities. The purpose of this study was to i) determine the relationship between SES and brain volume in regions susceptible to age related atrophy and, ii) ascertain whether the relationship was mediated by modifiable lifestyle factors.

## Methods

### Participants

The Coronary Artery Risk Development in Young Adults (CARDIA) is a longitudinal study which began in 1985–1986 when 5,115 participants (18–30 years old) were recruited by equal distribution of sex, age, education, and race with the aim to study determinants of cardiovascular disease in four U.S. cities (Birmingham, AL; Chicago, IL; Minneapolis, MN; and Oakland, CA [23]. Participants have completed numerous follow-up appointments with the most recent at the year 30 exam (2015–2016), when a subset of participants underwent magnetic resonance imaging (MRI) for the CARDIA Brain MRI Sub-study. This MRI sub-study was designed to characterize the morphology, pathology, physiology, and function of the brain in this cohort. The sample for the CARDIA Brain MRI sub-study were balanced within four strata of ethnicity/race (black, white) and sex from three of the CARDIA field centers (Birmingham, AL; Minneapolis, MN; and Oakland, CA).

### Standard protocol approvals, registrations, and patient consents

The MRI protocol was approved by the Institutional Review Board (IRB) of the participating sites including the University of Alabama Birmingham IRB, University of Minnesota IRB, Kaiser Permanente Northern California IRB, and the University of Pennsylvania IRB. Additional IRB approval was granted through the NIH Office of Human Subjects Research Protection for the Intramural Research Program, and the National Institute on Aging. All participants were provided and signed a separate written informed consent for the CARDIA Brain MRI Sub-study.

### Socioeconomic status

SES was operationalized as a composite score of total years of formal education and household income over the past 12 months. Because education and income are salient indicators of SES [6, 7] and national data demonstrates weak-moderate correlations between these measures [24], investigators routinely use a composite index of SES [3, 20, 25, 26]. Similar to previous research, education and income were categorized into approximately equal tertiles (education: 1 = ≤ 12; 2 = 13–16, 3 = ≥ 17; income: 1 = ≤ 49,999; 2 = 50,000–99,999; 3 = ≥ 100,000) and were summed to create a SES score which ranged from 2 (low SES) to 6 (high SES) [26].

### Neuroimaging protocol

The MRI scans were acquired in the axial plane on 3T scanners located at each CARDIA study sites; a Siemens 3T Tim Trio/VB 15 platform in Minneapolis and in Oakland; a Philips 3T Achieva/2.6.3.6 platform in Birmingham. Standard quality assurance protocols which were previously developed for the Functional Bioinformatics Research Network (FBIRN), and the Alzheimer’s disease Neuroimaging Initiative (ADNI) were used with the following thresholds: FBIRN—Siemens scanners Signal-to-Fluctuation-Noise-Ratio (SFNR) >220, Radius of Decorrelation (RDC)>3.1, Philips scanners SFNR>220, RDC>2.4; ADNI—Signal-to-Noise-Ratio (SNR) >300, Maximum Distortion >2.0. The structural images were acquired with 3D T1 and T2 sequences. Scan acquisition parameters have been previously described [27], and were processed using previously described methods [28–30]. In brief, structural images were processed using an automated multispectral computer algorithm which classified all supratentorial brain tissue into gray matter, white matter, and cerebral spinal fluid. After correction of intensity inhomogeneities [31] a multi-atlas skull stripping algorithm was applied for the removal of extra-cranial tissues [32]. Each T1-weighted scan is automatically segmented into a set of anatomical gray matter regions of interest (ROIs) using a mutli-atlas label fusion method, MUSE [33]. A total of 663 images were visually checked for incidental findings, motion artifacts, and other quality issues. For the current study, gray matter ROIs that correspond to the Spatial Pattern of Atrophy for Recognition of Brain Aging (SPARE-BA) index were chosen a priori due to these regions demonstrating early age-related atrophy patterns [14, 15]. These ROIs which include the insular cortex, thalamus, cingulate, frontal, inferior parietal, and lateral temporal cortex were summed together and then expressed as a percentage of intracranial volume (ICV) to account for differences in head size. The cerebellum was chosen as a control region.

### Questionnaire data

In addition to the MRI, all participants completed a variety of health-related questionnaires and measurements (e.g. height, weight) at the year 30 exam. Modifiable lifestyle factors that were investigated as potential mediating variables include physical activity, cognitive activity, smoking status, alcohol consumption, and diet. All exam materials (i.e. protocols, questionnaires) can be found on the public CARDIA website (https://www.cardia.dopm.uab.edu). *Physical activity* was measured by the CARDIA physical activity questionnaire. This interviewer-administered questionnaire inquires on vigorous (e.g. jogging, racket sports, heavy lifting on the job & sport), leisurely (e.g. non-strenuous sport, hiking, home exercising & gardening), and work-related (e.g. sitting frequency) activities over the previous year [34]. Scores were converted into total physical activity intensity scores which reflect the estimated number of kilocalories expended per activity, and expressed in exercise units [35]. *Cognitive activity* was assessed by the CARDIA Cognitive Activity Scale where participants rated how often they performed 10 cognitive activities (e.g. read newspapers, play cards) over the past 12 months. Each of the 10 cognitive activity items ranged from ‘never’ (0) to ‘daily’ (6) and were averaged into a total cognitive activity score (range: 0–6). *Smoking status* was determined by an interview questionnaire. The participants were classified as a ‘non-smoker’ (0), ‘former smoker’ (1), or ‘current smoker’ (2). *Diet* was measured based on a self-report question which inquired on the nutritional quality of their diet. Participants responded to one of four categories which ranged from low (1) to high (4) dietary quality (range: 1–4). *Alcohol consumption* was determined by an interviewer-administered questionnaire inquiring how many drinks (beer, wine, liquor) per week are typically consumed. The total number of drinks per week were then relativized (beer [16.7 mL/drink] + wine [17.0 mL/drink] + liquor [16.7 mL/drink]) into total milliliters of alcohol consumed. Median imputation was used to account for missing questionnaire data.

### Statistical analyses

Multivariable linear regression was used to test the association between SES and brain volume while adjusting for age, race and sex. Pearson correlations tested relationships between SES, brain volume, and modifiable lifestyle factors. Mediation analysis requires the following assumptions to be satisfied i) the independent variable (SES) must be associated with the dependent variable (brain volume), ii) the mediator variable (lifestyle factors) must be associated with the independent variable and the dependent variable, and iii) the mediator variable must significantly reduce the variance explained by the independent variable on the dependent variable [36]. After identification of potential mediators, our mediation analyses involved testing three regression models to determine whether the indirect effect of SES through modifiable lifestyle factors had a significant effect on brain volume [37]. Model 1 predicted brain volume from SES. Model 2 predicted lifestyle factors from SES. Model 3 predicted brain volume from both SES and lifestyle factors simultaneously. The Sobel test was used to determine whether the mediation effect was significant [38]. In brief, the Sobel test uses the beta coefficients and standard errors from the models to determine if the mediation effect on the dependent variable is significantly different than zero [37, 38]. The magnitude of mediation was determined by the percent reduction in the beta coefficient for SES predicting brain volume after inclusion of the mediating variable using the following formula: (β_Model 1_ − β_Model 3_) / (β_Model 1_) × 100. All statistical regression models were age, race, and sex adjusted with the significance level set at 0.05. Our statistical package was IBM SPSS, version 26.

## Results

A total of 645 participants (40.6% black; mean age 55.3 years ± 3.5) with processed and quality checked structural brain imaging data were included in the study. Brain volume was negatively associated with age (*r* = -.20; *p* < .001). The SES scores were normally distributed and the percentage of participants in each category is as follows: 2: 12%, 3: 22%, 4: 24%, 5: 30%, 6: 12%. Additional participant characteristics are listed in Table 1. The percentage of missing questionnaire data for each measured variable ranged from 1–3% and sensitivity analyses determined median imputation did not influence any of the reported findings (*p* >.05).

**Table 1:** Characteristics of study participants.

Variable	Entire Sample, n = 645	Low SES, n = 220	Moderate SES, n = 153	High SES, n = 272
Age, years	55.3 (3.5)	54.9 (3.7)	56.0 (3.2)	55.3 (3.5)
Sex, Female, %	52.9	51.4	52.9	54
Race, Black %	40.6	62.7	37.3	24.6
Education, years	15.1 (2.5)	13.1 (1.7)	14.9 (2.1)	16.9 (1.8)
Annual Income, dollars	69,364 (32,831)	33,480 (23,620)	73,608 (20,815)	96,002 (9,992)
SES, score	4.08 (1.2)	2.65 (0.46)	4.0 (0.0)	5.29 (0.45)
Brain Volume, SPARE-BA, % ICV	19.09 (1.2)	18.97 (1.3)	19.00 (1.2)	19.23 (1.1)
Physical Activity, total intensity score	344.3 (268.4)	284.3 (264.8)	331.9 (249.0)	399.9 (271.4)
Cognitive Activity, total	2.21 (.84)	1.89 (.88)	2.35 (.84)	2.40 (.72)
Smoking status, current %	12.1	25.9	9.2	2.6
Smoking status, former %	21.4	21.8	19.0	22.4
Smoking status, never %	66.5	52.3	71.9	75
Alcohol consumption, mL per week	12.80 (20.48)	12.87 (25.65)	11.43 (16.80)	13.51 (17.46)
Diet, score	2.65 (.74)	2.53 (.78)	2.70 (.71)	2.71 (.71)
Diabetes, % positive	9.5	11.4	8.5	8.5
Systolic blood pressure, mmHg	117.9 (15.2)	119.8 (16.6)	116.8 (14.1)	116.9 (14.4)
Diastolic blood pressure, mmHg	72.1 (10.8)	73.4 (11.6)	71.1 (9.4)	71.5 (10.7)
Body mass index, kg/m^2^	28.1 (4.7)	28.9 (5.1)	27.6 (4.4)	27.7 (4.6)

SES, Socioeconomic Status; Low SES, scores 2–3; Moderate SES, score 4; High SES, scores 5–6; Annual Income, median estimation from questionnaire; SPARE-BA, Spatial Pattern of Atrophy for Recognition of Brain Aging index; ICV, intracranial volume.

^†^ Values are represented as mean and standard deviation, unless otherwise indicated.

### SES and brain volume

**Fig 1:**
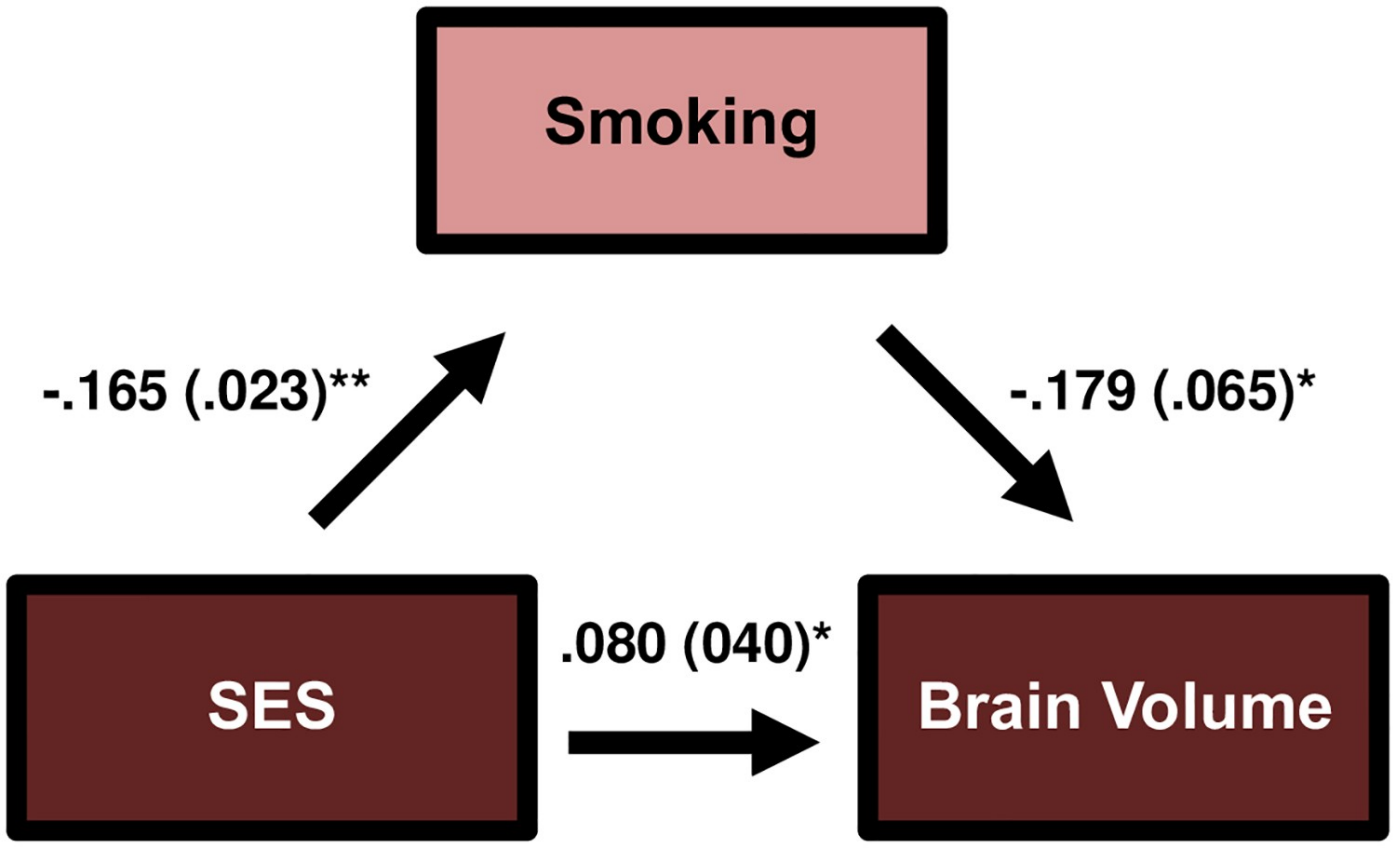
Smoking mediates SES and brain volume.

The overall regression model linking SES to brain volume was significant (F(4,640) = 25.75, *p* < .001). SES was significantly and positively associated with brain volume (β = .109 SE = .039; *p* < .01) while accounting for variance explained by age, sex, and race (Table 2). Having established that SES was significantly associated with brain volume, we proceeded determining whether this association was being mediated by any of the measured modifiable lifestyle factors (aim 2). To identify potential mediators, we investigated the individual relationships between each of the five lifestyle factors with SES and brain volume. Bivariate correlation analysis revealed all the measured lifestyle factors were correlated with either SES or brain volume (*p* < .05; Table 3), however smoking was the only variable negatively correlated with both SES (*r* = -.296, *p* < .001) and brain volume (*r* = -.128, *p* < .01). The Sobel Mediation analysis determined smoking status was a significant mediator between SES and brain volume (z = 2.57, *p* = .01; Fig 1). In the mediation model, smoking was negatively associated with SES (β = -.165 SE = .023) and brain volume (β = -.179 SE = .065) which consequently explained 27% of the variance initially attributed to SES with respect to brain volume (Table 4). There was no detected mediation with respect to SES and cerebellum volume.

**Table 2:** Multivariable linear regression Model 1: SES and brain volume.

Predictors	β (SE)	Standardized β	*p*-value
**SES**	**.109 (.039)**	**.112**	**.005**
Age	-.062 (.013)	-.179	< .001
Sex	.684 (.087)	.288	< .001
Race	-.201 (.098)	-.083	.040

SES, Socioeconomic Status; β, beta coefficient; SE, standard error.

^†^ Sex coded male (1) female (2); Race coded black (1) white (2).

**Table 3:** Bivariate correlations.

	Physical Activity, total intensity score	Cognitive Activity, total	Smoking, status	Diet, score	Alcohol consumption, mL per week
**SES, score**	.187**	.294**	-.296**	.134**	.022
**Brain Volume, SPARE-BA, % ICV**	.003	.064	-.128**	-.038	-.148**

SES, Socioeconomic Status; SPARE-BA, Spatial Pattern of Atrophy for Recognition of Brain Aging index; ICV, intracranial volume; Smoking Status coded never (0) former (1) current (2).

** Correlation is significant at the 0.01 level (2-tailed).

**Table 4:** Multivariable linear regression Model 3: SES, smoking, and brain volume.

Predictors	β (SE)	Standardized β	*p*-value
**SES**	**.080 (.040)**	**.082**	**.046**
**Smoking**	**-.179 (.065)**	**-.106**	**.006**
Age	-.061 (.013)	-.181	< .001
Sex	.680 (.087)	.286	< .001
Race	-.207 (.097)	-.086	.034

SES, Socioeconomic Status; β, beta coefficient; SE, standard error.

^†^Smoking coded never (0) former (1) current (2); Sex coded male (1) female (2); Race coded black (1) white (2).

## Discussion

In this population-based sample of biracial community dwelling middle-aged adults, SES was positively associated with brain volume in regions vulnerable to early age-related atrophy. Notably, we demonstrated smoking status significantly mediated this relationship, that is, lower SES was associated with greater smoking prevalence, which in turn had a negative effect on brain volume. We did not detect mediation from the other four modifiable lifestyle factors. This finding suggests smoking cessation may be a high yield target for early intervention to mitigate the adverse effect of low SES on brain health. In contrast, there was no observed effect with cerebellum volume, suggesting SES and smoking have specific effects with respect to gray matter volume in regions that are predictive of future cognitive impairment.

Low SES has been shown to be a risk-factor for dementia [6–10]. In a prospective study, Goldbourt and colleagues reported adults of low midlife SES were 3 to 6-times more likely to develop dementia compared to age-matched adults of higher SES standing [8]. These results echo those of prior studies that have reported a 2–3 fold increase in dementia prevalence for those of low SES [7, 9, 10], but there have been reported contradictions in the available literature [39]. Because brain atrophy becomes apparent in midlife [11] and this decline is predictive of future dementia [12], investigating whether SES is related to brain volume during middle-age may provide insight into potential neurobiological mechanisms through which high SES protects against future dementia. There is a growing body of research that has investigated SES and brain health in cognitively healthy adults [20–22]. Large scale studies (n ≥ 100) suggest higher SES in middle-late adulthood is a positive predictor of brain volume in several regions including the amygdala, hippocampus, cingulate, and temporal cortices [25, 40] (but see also [41]). Further, older adults of low SES have displayed accelerated rates of brain atrophy compared to those of higher SES [42]. Our findings from an epidemiological biracial middle-aged adult sample compliment and expand previous research by establishing a link between SES and brain volume within gray matter regions that have recently been shown to be sensitive to early age-related atrophy [14, 15].

The extant literature suggests SES may be associated with brain volume, yet these studies have not investigated modifiable lifestyle factors associated with SES, which may be mediating the observed associations. Because low SES is associated with a variety of unhealthy behaviors (e.g. poor diet, smoking, physical inactivity) [3–5] that have been previously shown to be associated with lower brain volume [14–16], the findings reported in the literature may at least partly be due to modifiable lifestyle factors. In the present study, all of the lifestyle factors investigated were significantly associated with either SES or brain volume (Table 3). However, only smoking was identified as a potential mediator due to significant relationships with both SES and brain volume. Mediation analysis determined smoking status significantly mediated the relationship between SES and brain volume, accounting for 27% of the variance from the observed association (Tables 2 and 4). This finding is in agreement with Stringhini and colleagues who examined the role of modifiable lifestyle factors (i.e. smoking, alcohol consumption, diet, and physical activity) in relation to SES and mortality. As expected, adults of low SES had significantly higher mortality rates than those of high SES. When lifestyle factors were investigated, smoking was the strongest mediator, accounting for 32% of the variance between SES and mortality risk [43]. Although smoking appears to be a salient contributor to the deleterious effects of low SES it is important to note that these SES disparities were still present after accounting for smoking with respect to mortality [43], and brain volume (Table 4). These findings compliment national data which describe targeting lifestyle factors alone is not sufficient to overcome the health-related disparities associated with low SES [44].

Similar to SES, smoking is a significant risk factor for dementia [45]. In population-based studies of adults cognitively healthy at baseline assessment, smoking significantly increased the risk of developing dementia compared to non-smokers [45, 46]. Further, existing data suggests that smoking alone may account for 10% of the dementia cases nationwide [18]. The specific physiological mechanisms underlying the increased risk of developing dementia for smokers are relatively unknown [47], however, the preponderance of data suggests a connection between smoking and brain volume. Smoking has been shown to negatively influence indices of neurovascular function such as cerebral perfusion [48] (i.e. blood flow) and white matter integrity [27], which may precipitate brain volume decline. Compared to non-smokers, smokers exhibit greater age-related whole brain and cortical volume loss [49], and the higher frequency of smoking accelerates this decline [50]. In agreement with the present study, smoking has been shown to be negatively associated with brain volume in several cortical regions within the SPARE-BA index including the thalamus, cingulate, insular, frontal and temporal cortices [50, 51]. In fact, research that applied the SPARE-BA index demonstrated smoking status was negatively associated with brain volume in a large epidemiological sample of ~3,000 adult participants [14].

In our sample, smoking was negatively associated with SES which parallels studies that report disproportionately high smoking rates in adults of lower SES [52]. Although smoking rates in the United States have declined over the past 50 years [53], there are clear smoking cessation differences among varying levels of SES. In a study that utilized the National Health and Nutrition Examination Surveys (NHANES), Kanjilal and colleagues investigated 30-year trends in smoking by SES levels among US adults. The authors reported a significant difference in smoking prevalence from years 1971–2002 among varying levels of SES, with the largest declines observed within the high SES group; the national averages decreased ~19%, whereas only modest declines were observed in the lowest SES group at ~6% [3]. Similarly, a Centers for Disease Control and Prevention (CDC) report which analyzed data from the 2008 National Health Interview Survey (NHIS) reported that from 1998–2008 adults of high SES had the greatest smoking cessation success compared to those of lower SES standing [52]. These data demonstrate individuals of lower SES have higher rates of smoking and are less likely to successfully abstain. A multitude of factors may contribute to the observed SES smoking disparity including access to treatment, social support, motivation, and life stressors [54]. With smoking prevalence disproportionately affecting those of lower SES, there is a need to identify successful strategies to promote cessation within SES disadvantaged populations.

Limitations of this study include its cross-sectional nature. Future studies would benefit from a prospective design to elucidate causality and directionality of the observed relationships between SES, smoking, and brain volume. Further, the modifiable lifestyle factors of interest were assessed through questionnaires, which are subject to recall and social desirability biases. For instance, physical activity was assessed via a self-report questionnaire which may be less sensitive than device measured physical activity (i.e. accelerometry). Due to the demographic make-up of the CARDIA Brain MRI Sub-study Cohort (i.e. ethnicity/race balanced from three CARDIA field centers) generalizability of our findings to other ethnic groups may be limited. Finally, all measures were collected at the year 30 exam period, therefore, we do not know whether changes in lifestyle factors over time have greater predictive value.

In summary, the present study provides evidence that within a biracial middle-aged cohort, smoking mediates the relationship between SES and brain volume within regions that are vulnerable to early age-related atrophy. This is an important finding as it is now well accepted that the neurobiology of dementia, including brain atrophy, begins during midlife [11–13], a period of aging where lifestyle factors can be altered to decrease one’s risk of future dementia [17–19]. While others have reported SES [21, 25, 40, 42] and smoking [14, 49–51] are each separately associated with brain volume, our findings suggest smoking is a significant contributor to the SES and brain volume relationship. Targeting this particular modifiable lifestyle factor may be an efficacious means to mitigate the deleterious effects of low SES on brain volume. This study contributes to research investigating mechanisms by which low midlife SES may increase the risk of future dementia, and further, what modifiable lifestyle factors may be targeted to mitigate SES associated health care disparities.
